# An efficient antenna system with improved radiation for multi-standard/multi-mode 5G cellular communications

**DOI:** 10.1038/s41598-023-31407-z

**Published:** 2023-03-13

**Authors:** Naser Ojaroudi Parchin, Heba G. Mohamed, Karim H. Moussa, Chan Hwang See, Raed A. Abd-Alhameed, Norah Muhammad Alwadai, Ahmed S. I. Amar

**Affiliations:** 1 School of Computing, Engineering and the Built Environment, Edinburgh Napier University, Edinburgh, EH10 5DT UK; 2 Department of Electrical Engineering, College of Engineering, Princess Nourah Bint Abdulrahman University, P.O. Box 84428, Riyadh, 11671 Saudi Arabia; 3 School of Internet of Things, Xi’an Jiaotong-Liverpool University, Suzhou, 215123 Jiangsu Province China; 4 Faculty of Engineering and Informatics, University of Bradford, Bradford, BD7 1DP UK; 5 Department of Physics, College of Engineering, Princess Nourah Bint Abdulrahman University, P.O. Box 84428, Riyadh, 11671 Saudi Arabia; 6 Department of Electronics & Communication, Air Defense College, Alexandria University, Alexandria, Egypt

**Keywords:** Electrical and electronic engineering, Information technology

## Abstract

This paper introduces a multi-input multiple-output (MIMO) antenna array system that provides improved radiation diversity for multi-standard/multi-mode 5G communications. The introduced MIMO design contains four pairs of miniaturized self-complementary antennas (SCAs) fed by pairs of independently coupled structures which are symmetrically located at the edge corners of the smartphone mainboard with an overall size of 75 × 150 (mm^2^). Hence, in total, the design incorporates four pairs of horizontally and vertically polarized resonators. The elements have compact profiles and resonate at 3.6 GHz, the main candidate bands of the sub-6 GHz 5G spectrum. In addition, despite the absence of decoupling structures, adjacent elements demonstrate high isolation. To the best of the authors’ knowledge, it is the first type of smartphone antenna design using dual-polarized self-complementary antennas that could possess anti-interference and diversity properties. In addition to exhibiting desirable radiation coverage, the presented smartphone antenna also supports dual polarizations on different sides of the printed circuit board (PCB). It also exhibits good isolation, high-gain patterns, improved radiation coverage, low ECC/TARC, and sufficient channel capacity. The introduced antenna design was manufactured on a standard smartphone board and its main characteristics were experimentally measured. Simulations and measurement results are generally in good agreement with each other. Moreover, the presented antenna system delivers low SAR with adequate efficiency when it comes to the appearance of the user. Hence, the design could be adapted to 5G hand-portable devices. As an additional feature, a new ultra-compact phased array millimeter-wave antenna with super-wide bandwidth and end-fire radiation is being introduced for integration into the MIMO antenna systems. As a result, the proposed antenna system design with improved radiation and multi-standard operation is a good candidate for future multi-mode 5G cellular applications.

## Introduction

The current generation (4G) wireless cellular systems are unable to meet future wireless communications requirements for high data rates. For these reasons, the 5th generation (5G) of wireless communications or mobile networks has been developed to address these challenges. It offers a variety of enhanced services for the internet of things (IoT), machine-to-machine (M2M), mobile broadband, massive MIMO, and ultra-reliable communications^1^. In order to acquire the main themes of 5G networks, MIMO systems with an increased number of radiation elements must be considered for future wireless networks^2,3^. MIMO technology with multiple antennas can significantly amend the reliability function^4,5^. It has been extensively used in 4G LTE and is expected to be widely used in 5G. MIMO technology not only can significantly improve the system reliability but also increase channel capacity without requiring extra power at both the transmitter and receiver ends^6,7^. The use of diversity schemes in MIMO antenna configuration is also considered to be a crucial component of combating fading and enhancing the reliability of wireless links by sending the same signals with uncorrelated antennas^8^.

In hand-portable smart devices, high-efficiency and low-profile antennas that offer sufficient bandwidth and mutual coupling characteristics are very suitable^9,10^. In addition, due to the limited available space on these devices such as smartphone boards, low-cost and compact planar microstrip antennas are an appropriate choice for cellular applications^11^. For sub 6 GHz 5G applications There have been many developments in smartphone antennas in recent years^12–21^. However, either these antenna arrays use resonators with single polarization, or they take up significant space on the mainboard. As smartphone PCBs are restricted in terms of antenna size, self-complementary antennas (SCAs) could be suitable for use in antennas, due to their compact size, simple structure, and ease of integration^22,23^. In addition, the SCA results in the geometry remaining unchanged when the metal and slot spaces are switched in a planar antenna^24^. We present here a new eight-port/four-antenna antenna array with miniaturized SCA radiators. In the single-element, the dual-polarized radiator has a self-complimentary structure and has been fed by independent coupled coaxial probes. The elements are highly miniaturized and resonate at 3.6 GHz. CST software package is used to design the antenna system^25^. Several unique characteristics distinguish this antenna from those reported in the literature, such as good isolation, high-gain patterns, excellent radiation coverage, low ECC/TARC, and sufficient channel capacity. Additionally, the proposed MIMO design is implemented, and its characteristics are analyzed. In order to verify the accuracy of the designed antenna performance, measurement results were carried out and the results were compared to electromagnetic simulations. Detailed descriptions of the double-fed SC resonator design and its array design are given.

Apart from sub 6 GHz frequencies, the MM-Wave spectrum is also expected to be supported by 5G smartphones^26^. In MM-Wave communications, phased array antennas with beam-steerable radiations are highly desirable since they enhance the radiation and connectivity of the systems. For smartphones, compact antennas can be used to form a linear phased array with high gain and directional radiation beams on the edges of the PCBs^27,28^. Moreover, end-fire antennas are more suitable for achieving the required full radiation coverage than conventional antennas, such as patch, slot, or monopole antennas^29^. Therefore, in addition to the proposed 3.6 GHz MIMO antenna, we have proposed a new mm-wave antenna package to operate at MM-Wave frequencies. The array consists of eight loop resonators arranged in a linear pattern, which can easily be integrated into smartphone antennas. The following sections present the design details, single-element performance, fundamental characteristics of the MIMO antenna system, and the suggested MM-Wave phased array, respectively.

## Dual-polarized SCA

**Figure 1:**
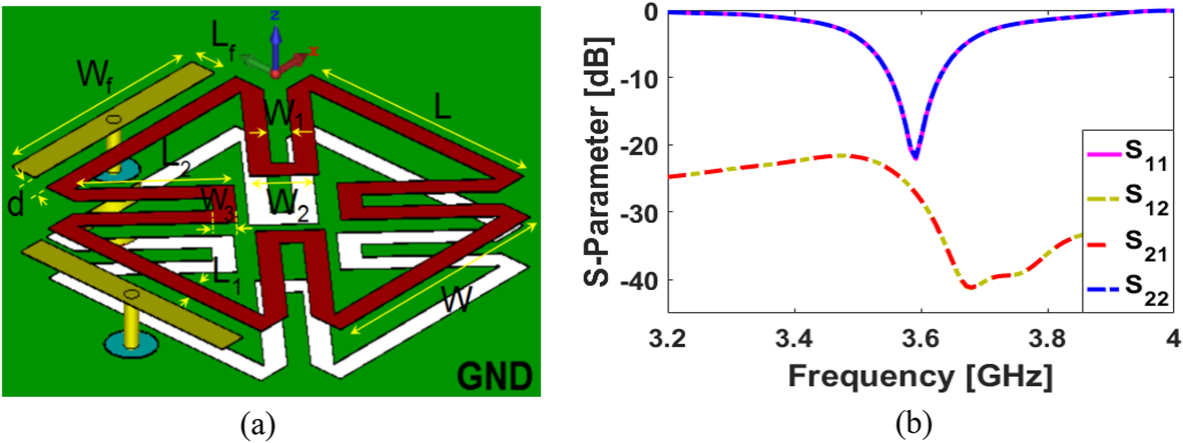
(**a**) Patch-slot antenna with double-fed self-complementary configuration and (**b**) its S-parameters.

Figure 1 shows the schematic diagram of the design. Figure 1a in which the petal-ring patch and slot structures are located on the top and back layers of the dielectric, respectively. Essentially, it consists of two independent coupled feeders connected to 50-Ohm coaxial probes. It is designed on a 1.6 mm Rogers-5880 dielectric material with 2.2 permittivity and the loss tangent of 0.0009. The antenna ground plane size is 15 × 15 mm^2^. Figure 1b shows the S parameters for the single SCA: It performs well within the preferred 3.6 GHz 5G band. As shown, more than 150 MHz bandwidth and better than − 20 dB mutual coupling have been discovered. However, prior to being miniaturized, the antenna underwent several evolutions. The resonator has a low profile of 10 × 10 mm^2^: its parameters (in mm) are listed in Table 1.

**Table 1:** Parameter values of the array design with SCA resonators.

Parameter	W	L	W_f_	L_f_	W_1_
Value (mm)	9.15	9.15	8.7	1	0.75
Parameter	L_1_	W_2_	L_2_	W_3_	d
Value (mm)	0.75	2.25	6.1	0.75	0.65

**Figure 2:**
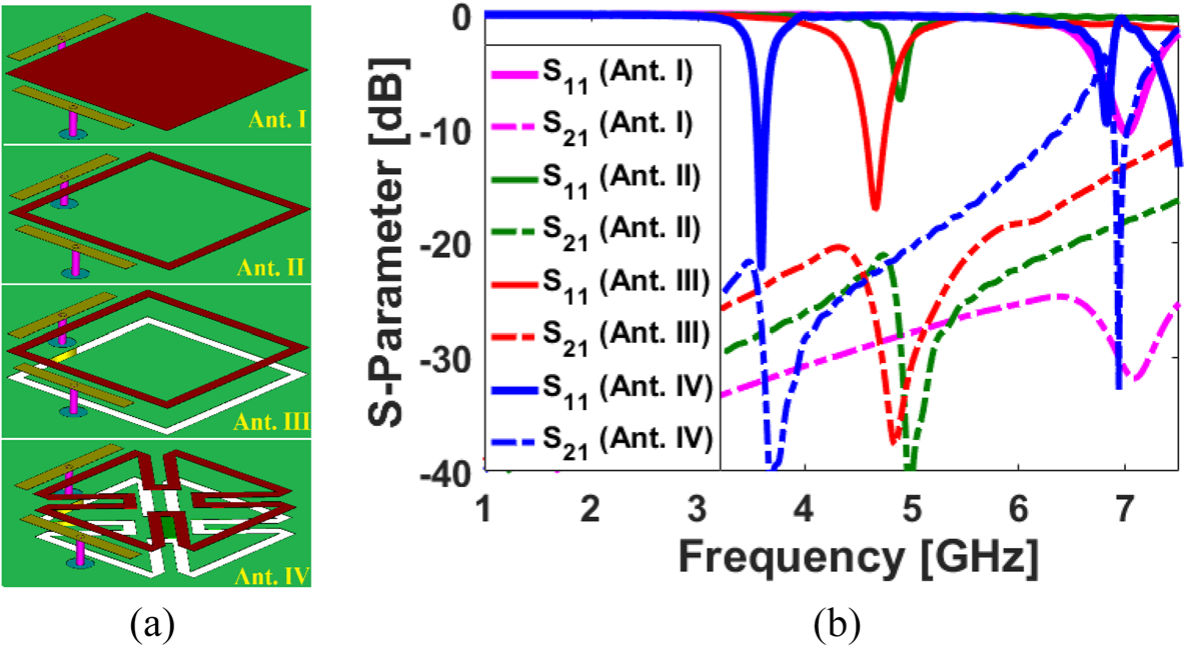
(**a**) Evaluation and (**b**) S-parameter investigation of SCA element.

The double-fed antenna element is depicted in Fig. 2 with its different configurations and S parameters. As shown, using the proposed design technique, the dual-polarized antenna’s operating frequency (from 7 to 3.6 GHz) can be significantly reduced without increasing the antenna’s overall size: the antenna’s operating frequency is reduced from 7 to 4.8 GHz by converting a square patch (Ant. I) to a square ring (Ant. II). Furthermore, cutting the complementary square-ring slot improves the antenna's matching and impedance bandwidth resonating at 4.6 GHz. Lastly, by converting the configuration of the SCA radiator, from square-ring (Ant. III) to petal-ring structure (Ant. IV), the electrical length of the radiator is increased, resulting in shifting the antenna frequency band from 4.6 to 3.6 GHz, which is a 5G candidate band.

**Figure 3:**
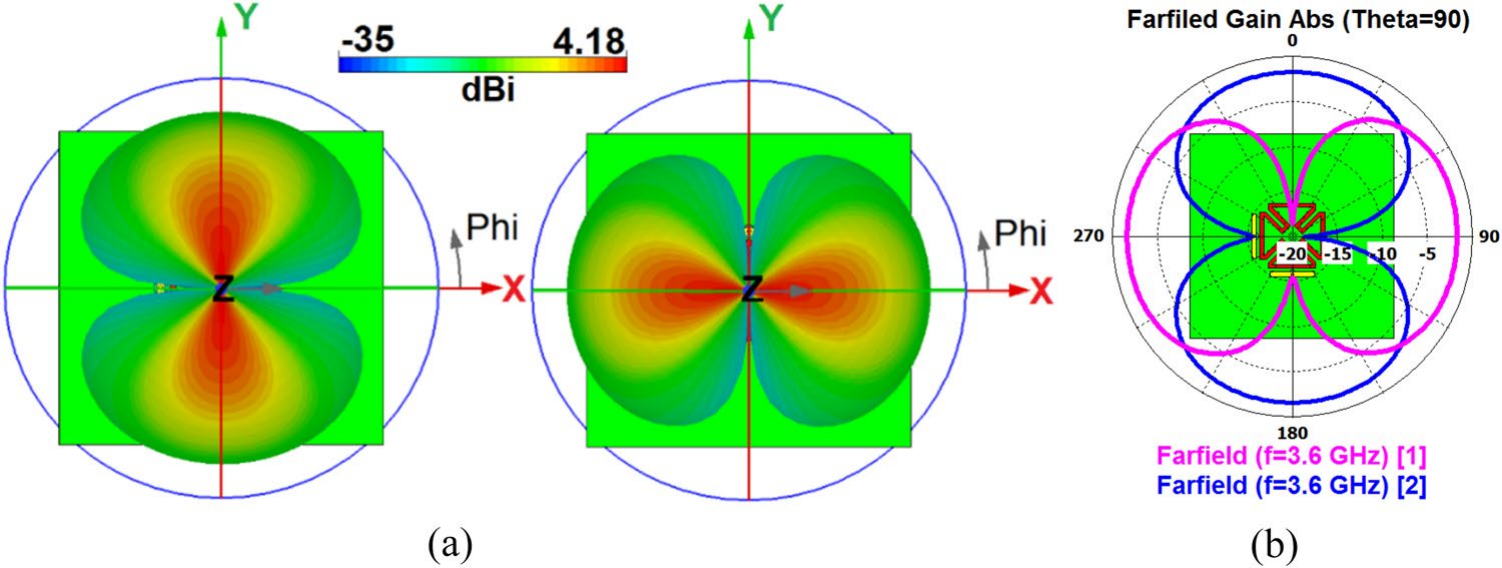
(**a**) 3D and (**b**) 2D radiations.

Figure 3a shows the radiation patterns (Phi) for the different antenna ports. It is shown that the dual-polarized antenna provides identical radiations and more than 4.1 dBi IEEE gain. As illustrated in Fig. 3b, similar radiation performances with a 90° difference and dual polarizations are observed due to different placements of the feeding ports^9,30^. The antenna configuration is flexible and different feeding techniques such as aperture coupling, microstrip line, and coaxial feedings can be applied. However, based on simulations, the employed feeding technique, which combines the coaxial and coupling feeds, offers a better performance in terms of impedance matching, bandwidth, and isolation. Moreover, since the elements are placed at the corners, the employed feeding technique is more appropriate to reduce the occupied space in the mainboard^31^.

**Figure 4:**
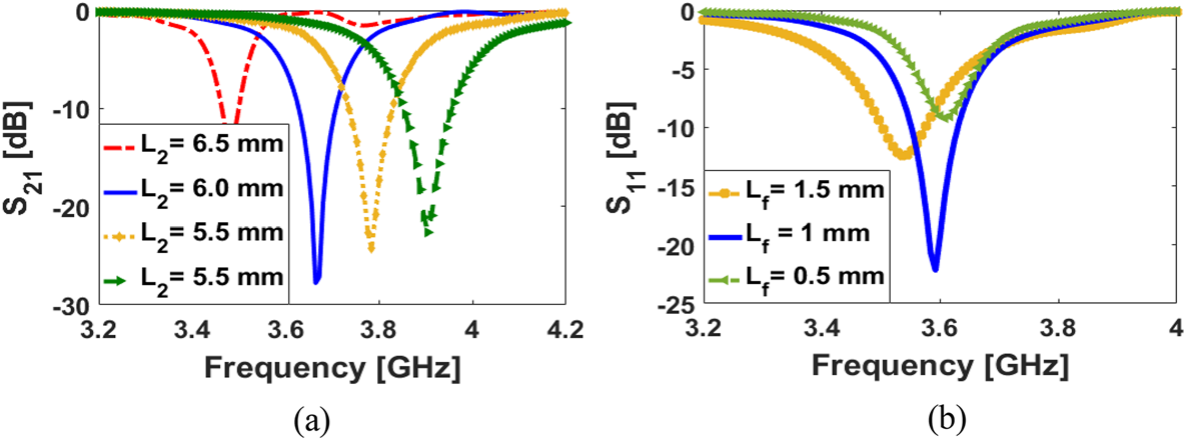
S_11_ results for various sizes of (**a**) L_2_ and (**b**) L_f_.

**Figure 5:**
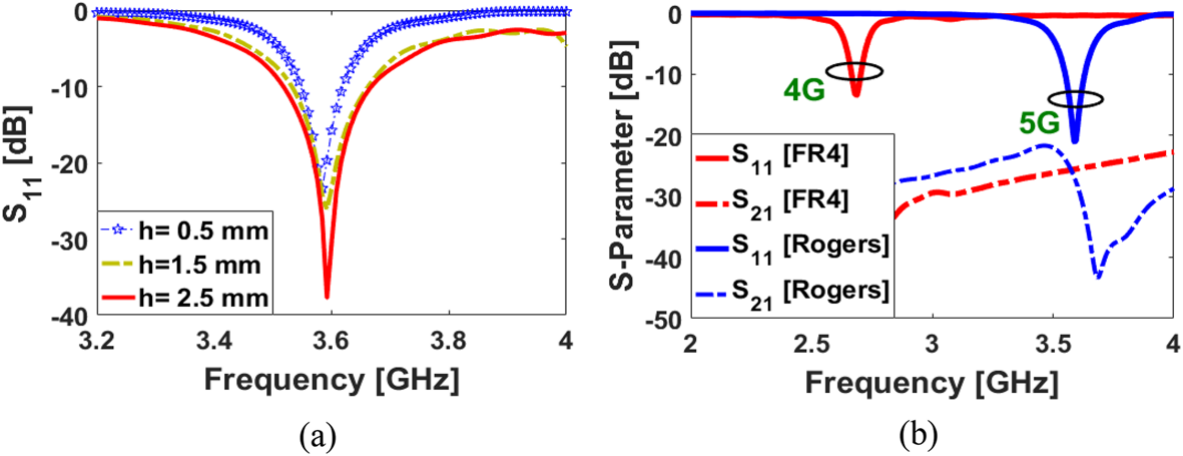
S_11_ results for various sizes of (**a**) h, and (**b**) the substrate types.

**Figure 6:**
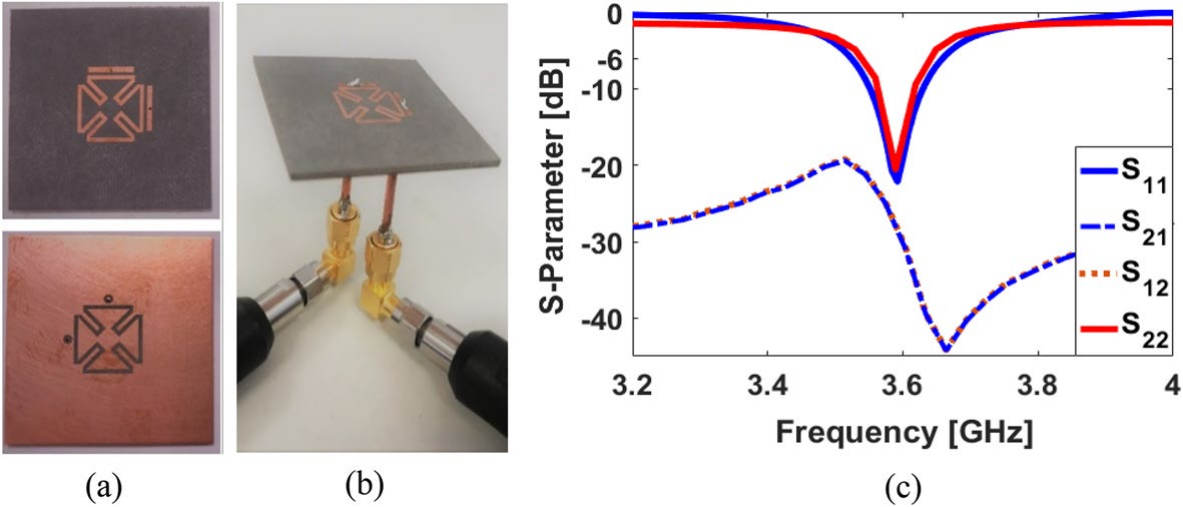
(**a**) Fabricated sample, (**b**) feedings, and (**c**) the measured S-parameters.

The frequency response of the SCA can be easily changed and tuned to the desired band, by changing the design parameters. An important parameter to consider relates to the arm of the petal-ring structure (*L*_2_). The S_11_ results of the dual-polarized SCA with various sizes of *L*_2_ are shown in Fig. 4a: when the length of the petal-ring arms decreases from 6.5 to 5 mm, it is possible to tune the resonance frequency from 3.5 to 3.9 GHz while maintaining similar impedance matching. The discussed antenna performance is also affected by the size of a pair of independent coupled feeding structures. The S_11_ results of the SCA with various values of L_f_ are represented in Fig. 4b. As illustrated, depending on the value of the coupled feeding structure, the single element’s operating frequency can vary. Another important parameter of the dual-polarized design is the thickness of the substrate. It can affect the impedance matching and the bandwidth of the SCA. Figure 5a illustrates the S_11_ results of changing substrate thickness (h): increasing the substrate thickness allows good impedance matching with a wider bandwidth. The proposed design has also the potential to be used for 4G applications. The S-parameters for different types of substrates are shown in Fig. 5b. As seen, the antenna with FR-4 dielectric can operate around 2.6 GHz (4G LTE operation band) and with the Rogers substrate, it works in the 3.6 GHz 5G band^32^. A prototype antenna has been fabricated and tested. The fabricated sample and its measured S parameters are shown in Fig. 6. As shown, the measured results of the fabricated prototype align well with simulations and appear to work correctly (Fig. 6c).

### Smartphone antenna array

**Figure 7:**
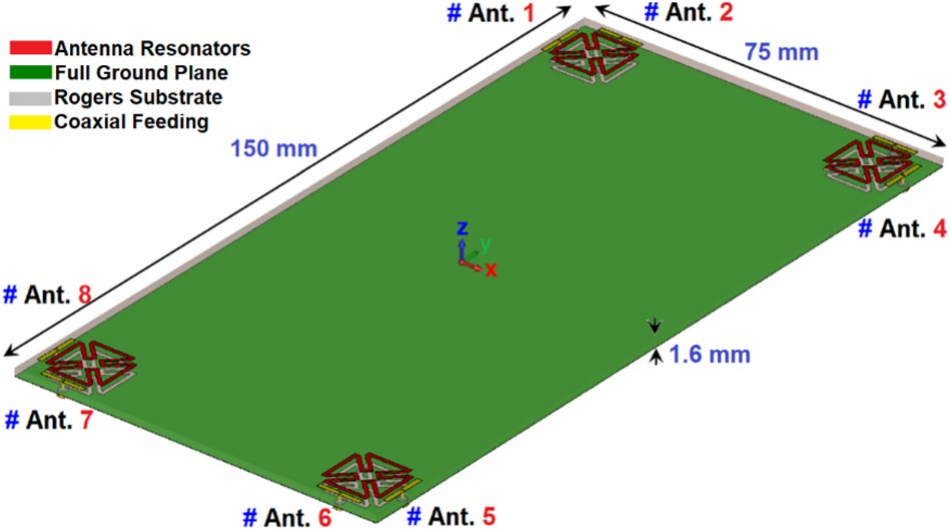
The MIMO smartphone array schematic.

Figure 7 shows the proposed MIMO design in a perspective 3D side view. As shown, the multi-feed antenna system has a rather straightforward and simple structural configuration. The overall size is 75 × 150 mm^2^ and the antenna elements are self-complementary resonators with the coaxial feeding method. At each edge of the smartphone mainboard, similar patch-slot antenna elements with dual polarizations are placed. By employing the complementary slot structures below each patch radiator, not only the matching function but also the radiation coverage of the system along the top and bottom of the board has been improved^33^.

**Figure 8:**
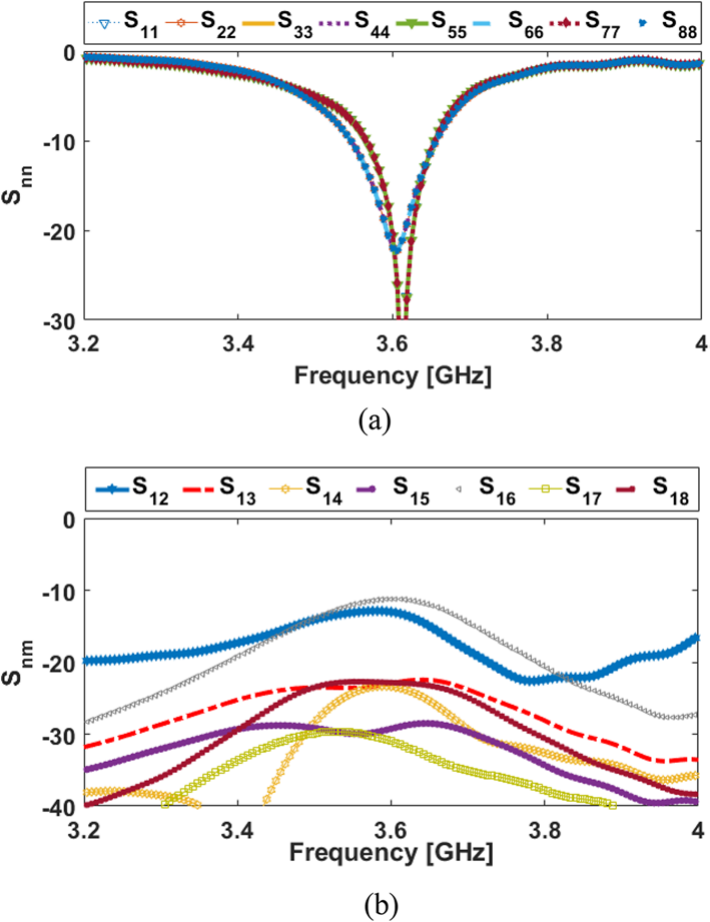
The simulated S-parameters for different elements: (**a**) S_nn_ and (**b**) S_nm_.

**Figure 9:**
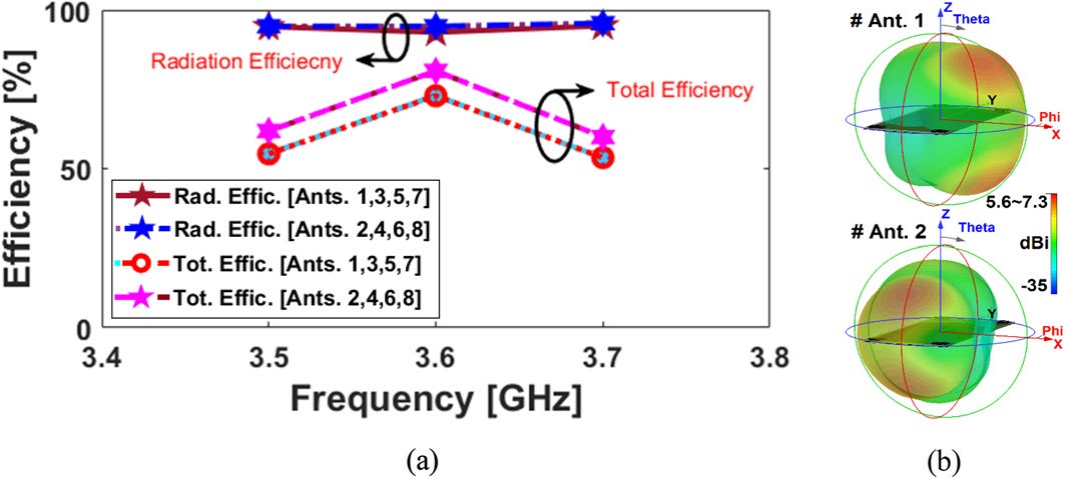
(**a**) Efficiencies of the MIMO elements, and (**b**) radiation patterns of Ant. 1 & Ant. 2.

The S-parameters for the antenna array shown in Fig. 8 indicate that the antenna elements provide almost identical frequency responses with high matching and less than − 20 dB reflection coefficients (S_nn_) at 3.6 GHz. In addition, as shown in Fig. 8b, the antenna elements have good isolation and less than − 12 dB mutual coupling (S_nm_). Based on the simulation, sufficient efficiency properties were obtained, as shown in Fig. 9a. From the figure, it is clear that all antennas have high radiation efficiency, exceeding 95%. A total efficiency of more than 70% is also demonstrated for each element at the resonance frequency of 3.6 GHz. Furthermore, as represented, for the range of 3.5–3.6 GHz, fairly acceptable simulated efficiencies, sufficient for MIMO smartphone operations have been observed.

**Figure 10:**
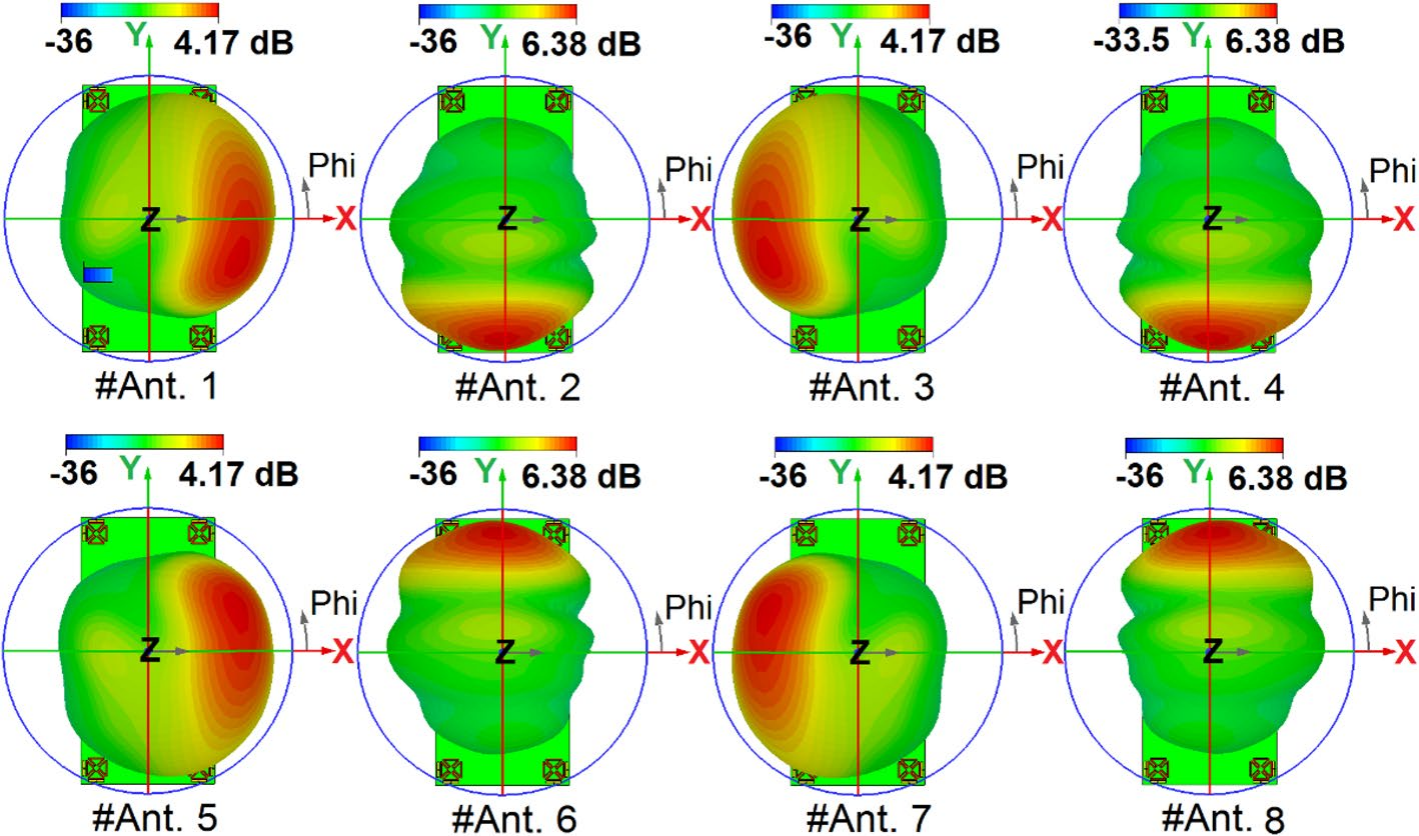
3D radiation patterns with realized-gain levels for Ant.1 ~ Ant.8.

The broadside radiations of a single-element radiator with different polarizations are plotted in Fig. 9b. Clearly, the resonator provides high directivities and symmetric radiations which improves the radiation coverage^34^. To have a better view, an illustration of the radiation patterns and gain values of the elements are given in Fig. 10. As seen, offering dual-polarizations and high-gain radiations for different regions of the required coverage through the eight antenna elements^35^. Therefore, the MIMO array design could be robust to the various holding positions of 5G smartphones.

**Figure 11:**
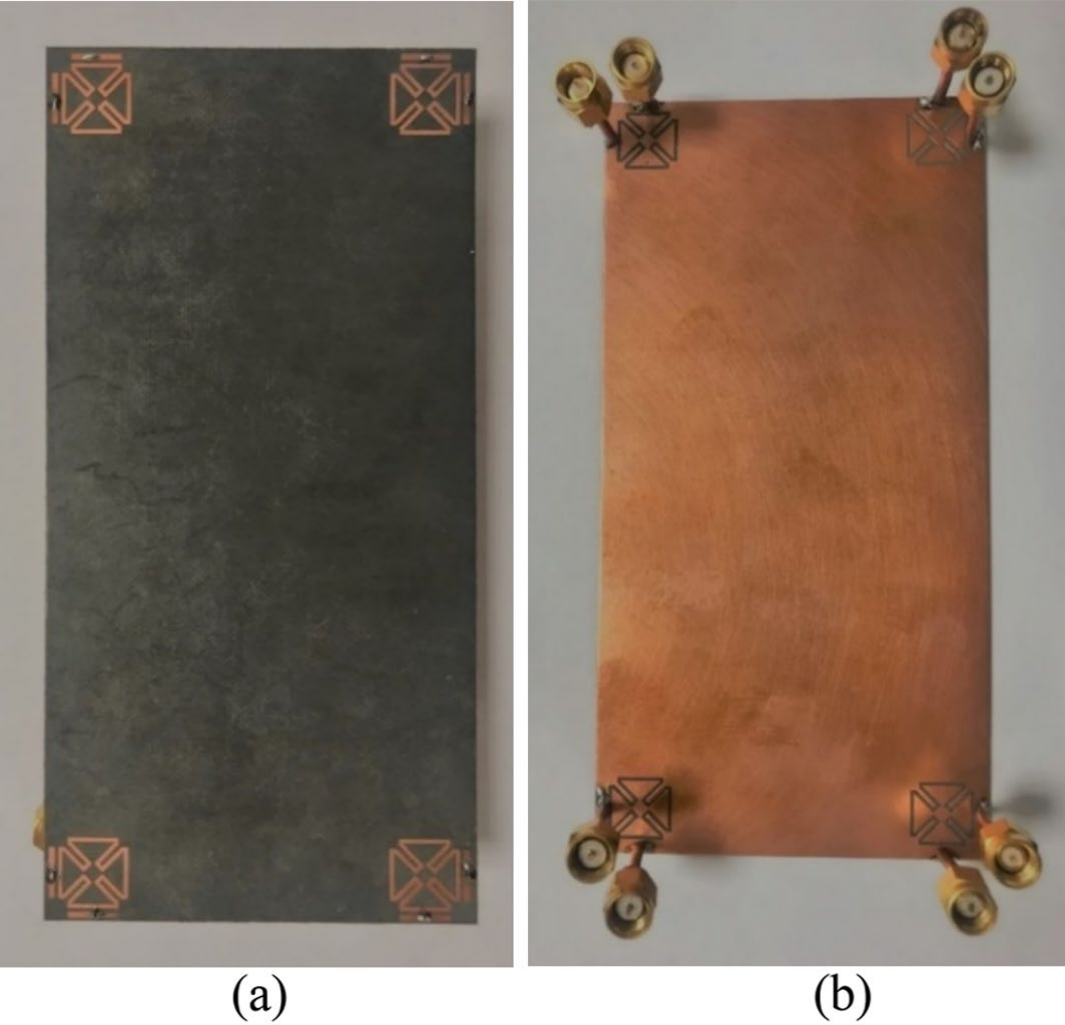
(**a**) Front, (**b**) back schematics of the fabricated MIMO smartphone design.

**Figure 12:**
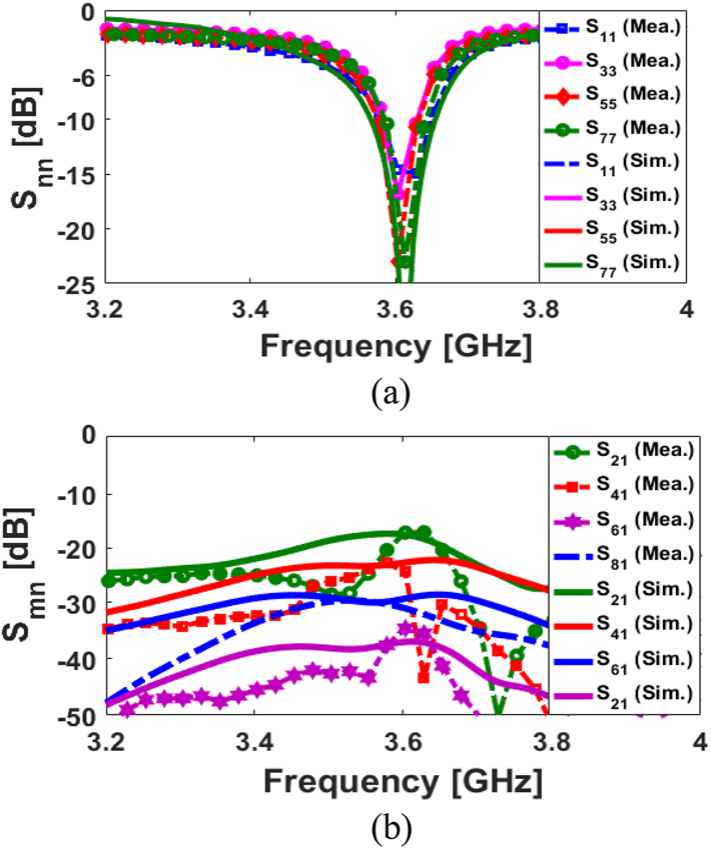
Comparison of the measurements and simulations for (**a**) S_nn_ and (**b**) S_mn_ characteristics.

A prototype of the proposed MIMO design has been developed and tested. The photos of the prototype sample (front/back layers with connectors) are shown in Fig. 11a,b. Figure 12a,b illustrate the reflection/transmission coefficients (S_nn_/S_mn_): well-defined results are obtained for SCA resonators. Additionally, the measurements are in good agreement with the simulations, indicating sufficient − 10 dB bandwidth and low couplings. A very slight variation was observed, possibly due to the errors in prototyping, feeding of antennas, and experimental setup. In addition, the S_21_ characteristics differ slightly from single antennas because of the large ground plane of the main design.

**Figure 13:**
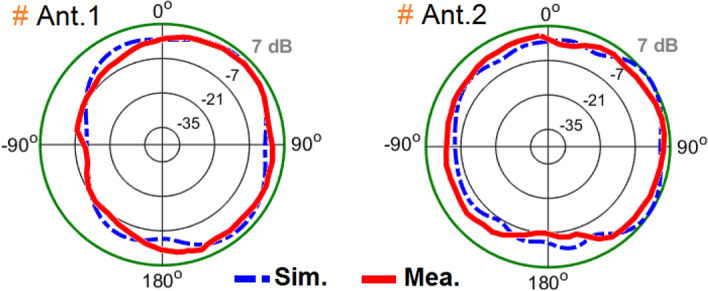
Measured and simulated data of two antenna elements’ radiations (E-Plane).

As the antenna pair’s performances were identical, radiations from the adjacent SCA radiators (Antennas 1 and 2) at 3.6 GHz were measured and plotted in Fig. 13. It can be seen that the fabricated prototype exhibits desirable radiation, which corresponds well with the simulations. In addition, the corresponding elements provide high gains.

ECC (envelope correlation coefficient) and TARC (total active reflection coefficient) properties of the presented array are examined in order to verify its capability in MIMO operation and considered in the following^36^. It is worth mentioning that these parameters have been computed using the below formula:

1 $$ECC=\frac{{\left|{S}_{mm}^{*}{S}_{mn}+{S}_{nm}^{*}{S}_{nn}\right|}^{2}}{{\left(1-{\left|{S}_{mm}\right|}^{2}-{\left|{S}_{mn}\right|}^{2}\right)\left(1-{\left|{S}_{nm}\right|}^{2}-{\left|{S}_{nn}\right|}^{2}\right)}^{*}} ,$$

2 $$TARC=-\sqrt{\frac{{\left({S}_{mm}+{S}_{mn}\right)}^{2}+{\left({S}_{nm}+{S}_{nn}\right)}^{2}}{2}.}$$

**Figure 14:**
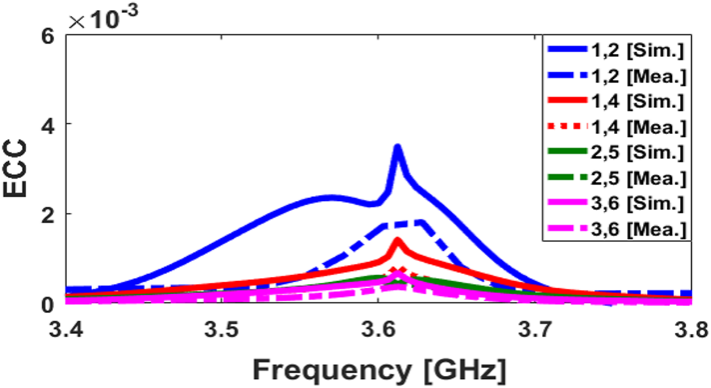
Calculated simulated/measured ECC results.

**Figure 15:**
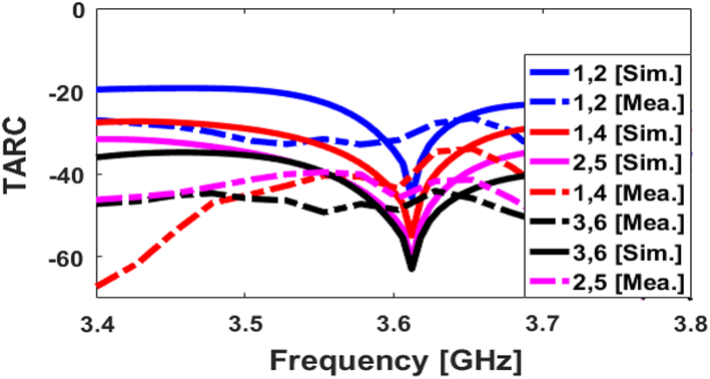
Calculated simulated/measured TARC results.

Figures 14 and 15 illustrate the calculated ECC and TARC properties, respectively. As shown, the ECC/TARC results are quite low (less than 0.004 and − 30 dB at 3.6 GHz, respectively) in the target frequency band. Another factor function which evaluates the MIMO performance and pattern diversity is the channel capacity loss (CCL) which can be achieved from the mutual correlation of the array elements^37^. In addition, for further investigation of the MIMO performance across the band of interest, the computed channel capacity (CC) is also studied^38^. The CCL and CC are defined as follows:

3 $$\text{CL}=-{\text{log}}_{2}\text{det}\left[\begin{array}{ccc}{\uprho }_{11}& \cdots & {\uprho }_{18}\\ \vdots & \ddots & \vdots \\ {\uprho }_{81}& \cdots & {\uprho }_{88}\end{array}\right],$$

4 $${\text{CC}} = {\text{E}}\left\{ {\log _{2} \left[ {\det \left( {{\text{I}} + \frac{{{\text{SNR}}}}{{{\text{n}}_{{\text{T}}} }}} \right){\text{H}}_{{{\text{scale}}}} {\text{H}}_{{{\text{scale}}}}^{{\text{T}}} } \right]} \right\},$$

where ${\uprho }_{\text{ii}}=1-({\left|{\text{S}}_{\text{ii}}\right|}^{2}+{\left|{\text{S}}_{\text{ij}}\right|}^{2})$, ${\uprho }_{\text{ij}}=-({\text{S}}_{\text{ii}}^{*}{\text{S}}_{\text{ij}}+{\text{S}}_{\text{ji}}^{*}{\text{S}}_{\text{ij}})$ and H_scale_ is the channel matrix.

**Figure 16:**
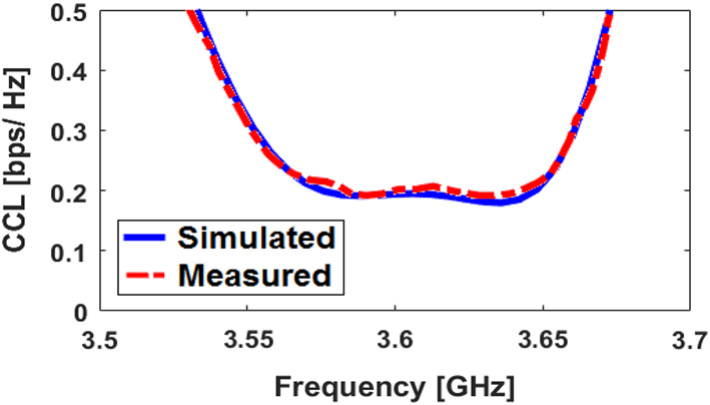
Calculated CCL function for the corresponding array.

**Figure 17:**
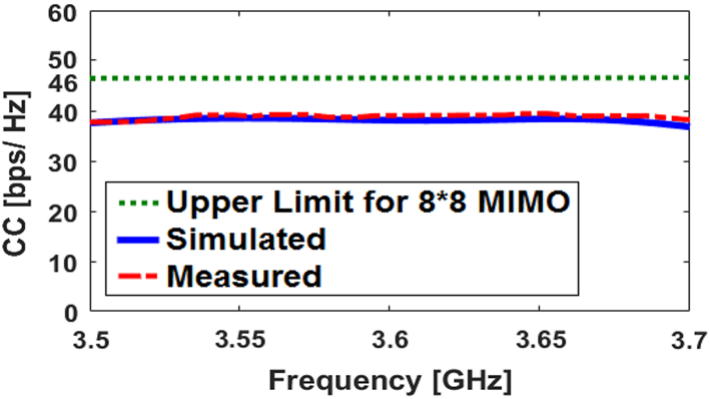
Calculated channel capacity for the corresponding array.

Figures 16 and 17 depict the calculated corresponding CCL and CC results, respectively. As plotted in Fig. 16, the array design has very low CCL which is appeared less than 0.5 bps/Hz) over entire its impedance bandwidth. Moreover, as indicated in Fig. 17, the calculated CC of the design is about 40 bps/Hz.

**Figure 18:**
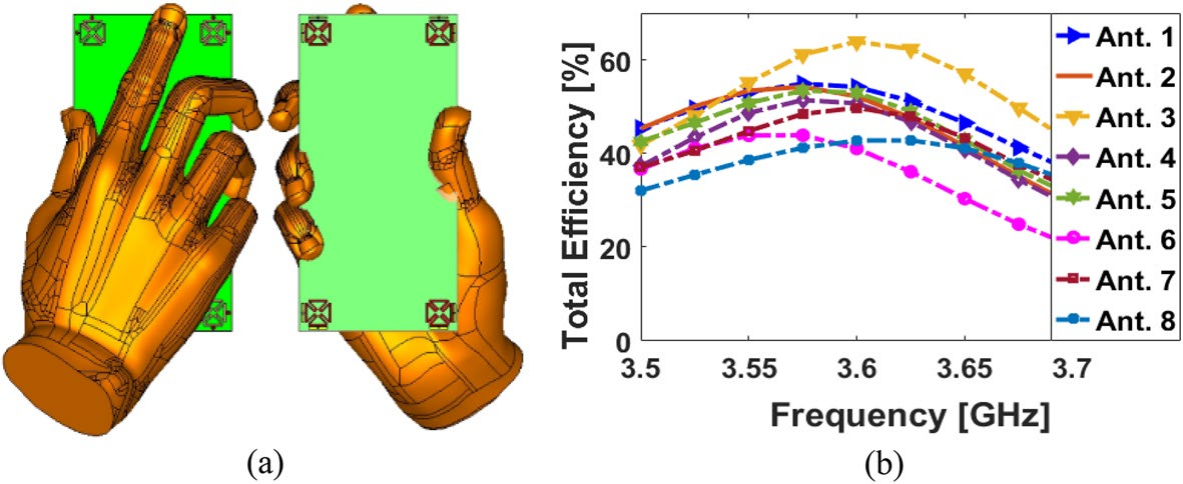
(**a**) Placement of the antenna and (**b**) the achieved efficiencies.

**Figure 19:**
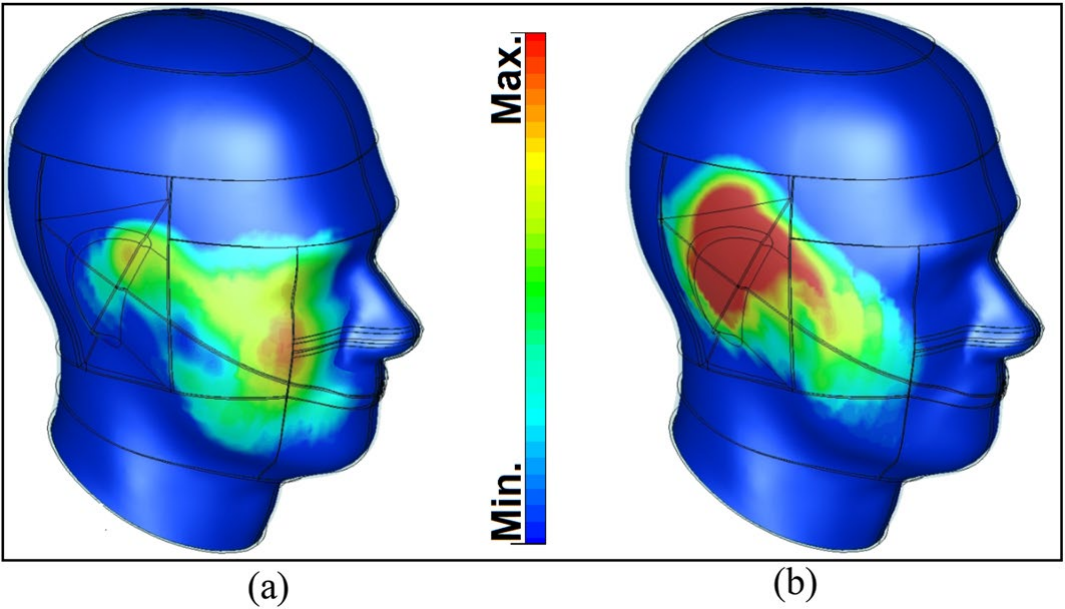
Investigated SAR impacts from (**a**) Antenna 3 and (**b**) Antenna 7.

This Part of this study discusses the user effects as it pertains to antenna performance and SAR levels in appearances of hands and head phantoms. Figure 18 shows the MIMO array placements and total efficiency results in the appearance of the user (hand phantom) with ε = 24 (permittivity) and σ = 2 s/m (conductivity)^39^. Based on the results shown, the proposed antenna design exhibits adequate performance. A careful investigation reveals that the resonators partially surrounded by the hand phantom suffer the greatest radiation losses. In most cases, this is due to the nature of the tissue, since it usually absorbs radiation power^40,41^. The specific absorption rate (SAR) distribution for the elements (including Antennas 3 and 7) is investigated and shown in Fig. 19. As shown, it is found that Antenna 3 contributes 1.8 dB (W/kg), the highest SAR value, whereas lowest SAR value (0.6 dB (W/kg)) is observed from Antenna 7. Due to the array arrangement, Antenna 3 appears to be located closer to the head-phantom, compared to Antenna 7. As a result, closer distances between resonators and phantoms contribute to the highest SAR levels, and vice versa.

**Figure 20:**
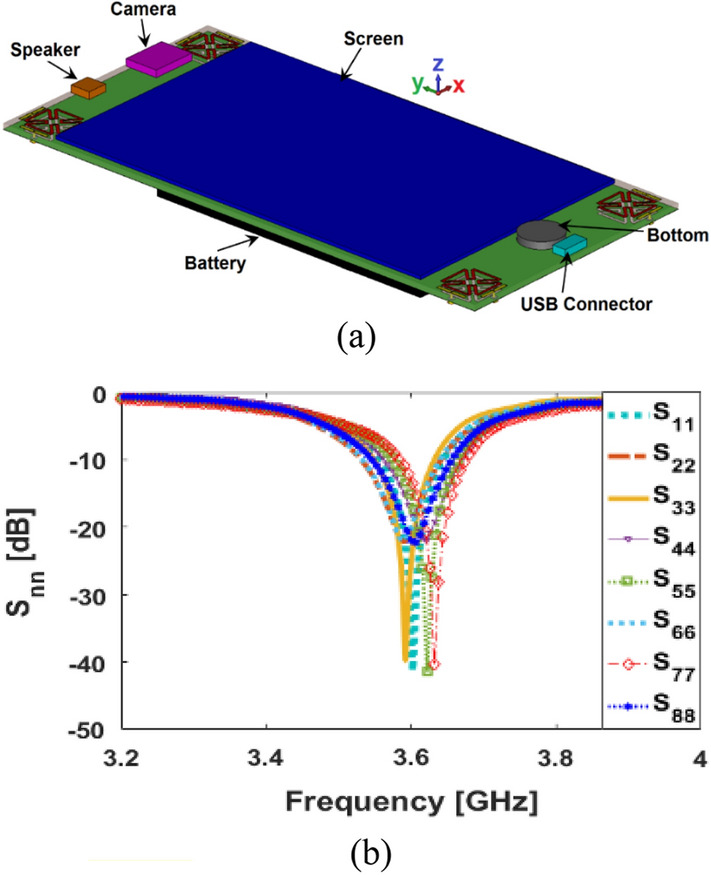
(**a**) Configuration and (**b**) reflection coefficients of the antenna module integrated with the components.

The S_nn_, reflection coefficient (S_11_ to S_88_) results of the MIMO smartphone array structure in the presence of various integrated components (including speaker, camera, LCD, battery, and USB connector) are represented in Fig. 20. It has been discovered that the MIMO antenna design has been found to operate around 3.6 GHz bands with a return loss lower than − 20 dB. Based on the illustration in Fig. 20b, the variations of the resonances are insignificant.

The performance comparison between the introduced MIMO antenna and the reported design in the literature is represented in Table 2. Various fundamental properties are discussed, including the type of element, the efficiency/gain results, and the ECC function. It has been discovered that the developed antenna array has shown improved performance with sufficient characteristics. Unlike most reported sub-6 GHz 5G antenna designs, the proposed design is planar, easy to integrate, and offers full radiation coverage on different board sides. Additionally, it provides high antenna gains and radiation/total efficiency. Aside from the array design's desirable performance, it also exhibits desirable performance when combined with the smartphone components and the user-phantom.

**Table 2:** Comparison Table of the introduced MIMO smartphone antenna.

Ref.	Antenna type	Frequency (GHz)	Efficiency (%)	Gain (dBi)	Size (mm^2^)	Isolation (dB)	ECC	Radiation diversity
^12^	Coupled monopole-slot	2.6 GHz (2.55–2.65)	50–70	3	136 × 68	12	< 0.15	Yes
^13^	Fractal monopole	3.5 GHz (3.4–3.6)	65–75	–	150 × 75	12	< 0.15	No
^14^	L-shaped strips	3.5 GHz (3.4–3.6)	50–5	4	136 × 68	15	< 0.1	No
^15^	I-shaped grounding	4.5 GHz (3.7–5.5)	–	4.5	140 × 68	15	< 0.40	Limited
^16^	Self-isolated element	3.5 GHz (3.4–3.6)	60–70	–	150 × 75	19	< 0.02	No
^17^	Balanced open-slot	3.5 GHz (3.4–3.6)	60–75	3.5	150 × 80	17	< 0.05	No
^18^	Communal square-loop	2.6 GHz (2.55–2.65)	45–60	2.5	136 × 68	12	< 0.20	Yes (Limited)
^11^	Inverted-F antennas	3.5 GHz (3.4–3.6)	–	4	110 × 60	19	–	Yes (Limited)
^19^	Quad-antenna linear (QAL)	3.5 GHz (3.4–3.6)	40–60	–	150 × 75	12	< 0.4	No
^20^	Loop resonators	3.5 GHz (3.45–3.55)	35–50	2	145 × 70	16	< 0.2	No
^21^	Open-end slot	3.5 GHz (3.4–3.6)	50–60	4.8	136 × 68	11	0.05	No
^34^	Coaxial-fed patch	4.7 GHz (4.4–5)	40–80	6	150 × 80	12	< 0.2	No
^39^	Meandered-dipoles	3.5 GHz (3.45–3.55)	48–67	–	144 × 74	15	< 0.10	No
^40^	Dual-feed module	3.5 GHz (3.45–3.55)	50–68	6.5	130 × 50	15	< 0.3	No
^41^	Monopoles	4.65 GHz (4.55–4.75)	50–70	1	136 × 68	10	–	Yes (limited)
This work	Miniaturized diversity SCA	3.5 GHz (3.5–3.7)	50–80	4.5–7	150 × 75	16	< 0.03	Yes full-coverage

## Modified smartphone antenna design with full ground plane

**Figure 21:**
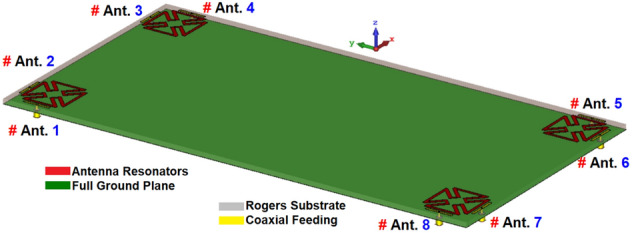
The array with full ground plane.

**Figure 22:**
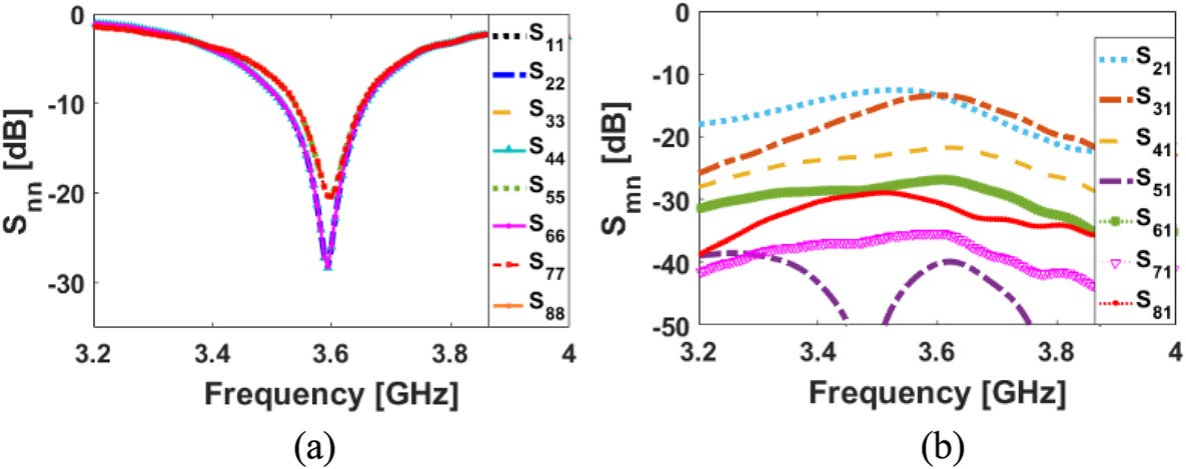
(**a**) S_nn_ and (**b**) S_mn_ results of the smartphone antenna with full GND.

**Figure 23:**
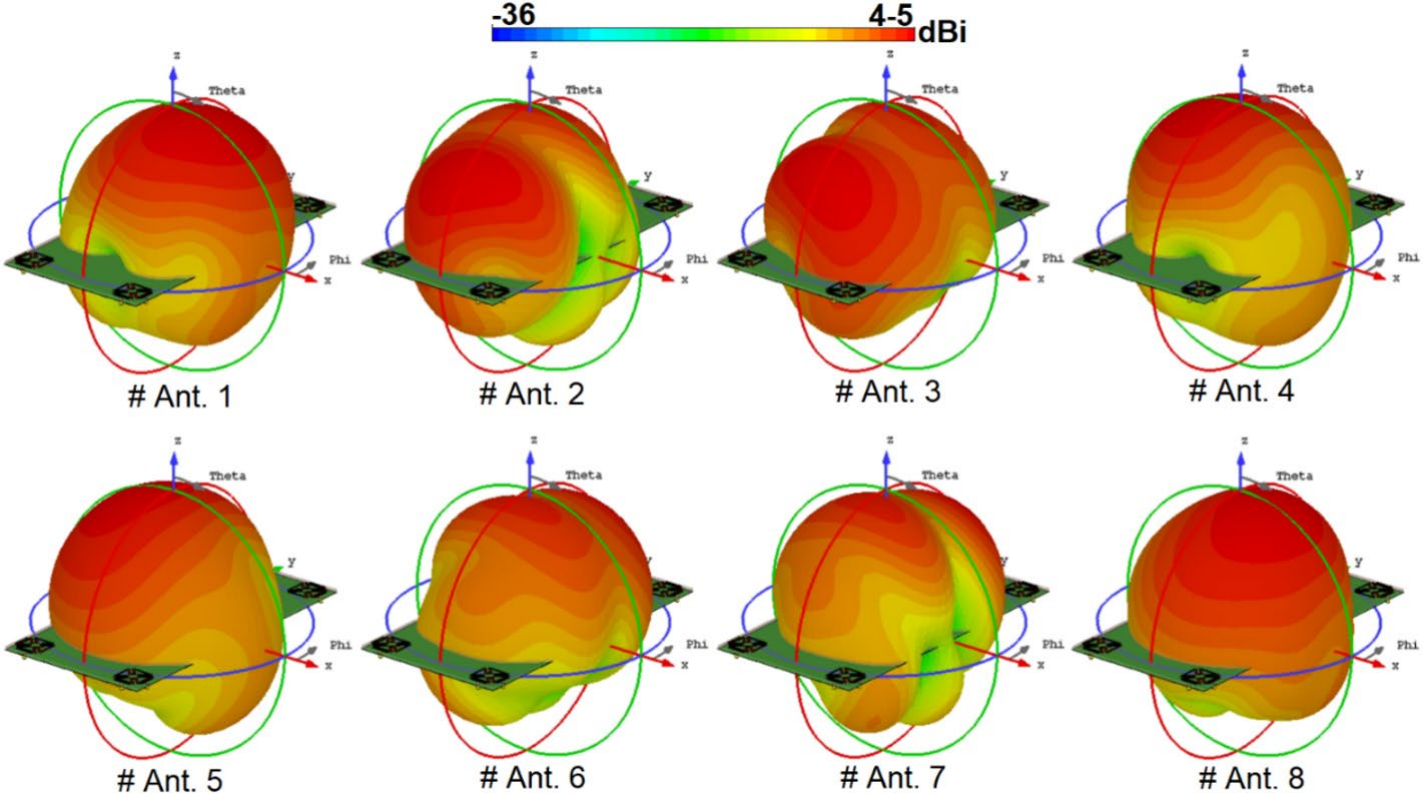
Typical radiation patterns for the modified GND MIMO antenna at 3.6 GHz.

This section examines the performance of dual-polarized MIMO antennas with a full ground (GND). Modifying the configurations and increasing the sizes of dual-polarized patch radiators without complementary slots in the ground plane enables the proposed smartphone antenna to operate at 3.6 GHz. In this case, it is necessary to modify the parameter values of a single-element patch-ring resonator as follows (in mm): W = 14.5, L = 14.5, W_f_ = 8.7, L_f_ = 0.8, W_1_ = 1, L_1_ = 0.85, W_2_ = 3, L_2_ = 8, W_3_ = 0.85, d = 0.6. The schematic for the modified design is shown in Fig. 21. S-parameters for the full GND design are shown in Fig. 22. As can be seen, the antenna elements provide sufficient S_nn_ and better than − 10 dB S_mn_ at 3.6 GHz, which is the 5G band. However, as seen in Fig. 23, since the antenna elements can only radiate through the top side of the PCB, the modified MIMO antenna design has limited radiation coverage. This is mainly due to lack of the slot resonators in the ground plane. Therefore, it can be concluded that the employed slots in the ground plane of the original design play a very vital role in improving the radiation coverage of the smartphone antenna^42^.

## Integration of a low-profile/super-wideband MM-wave phased array

**Figure 24:**
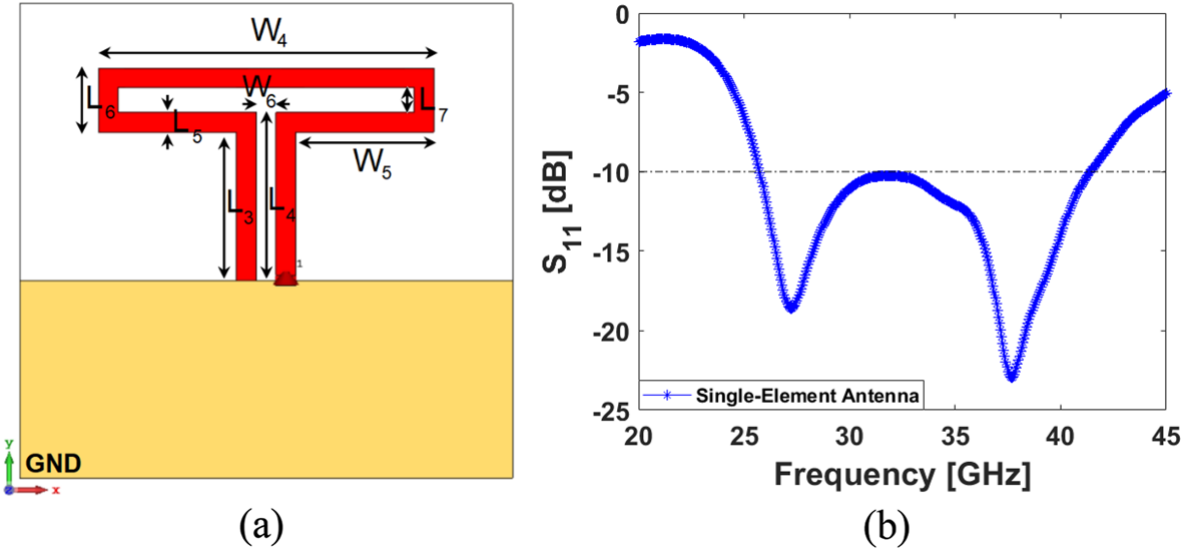
(**a**) Single-element loop resonator configuration and (**b**) its S_11_ result.

**Figure 25:**
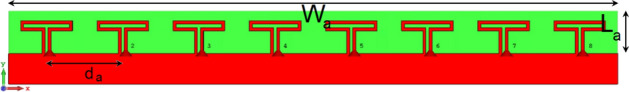
The schematic of the eight-element linear phased array.

**Figure 26:**
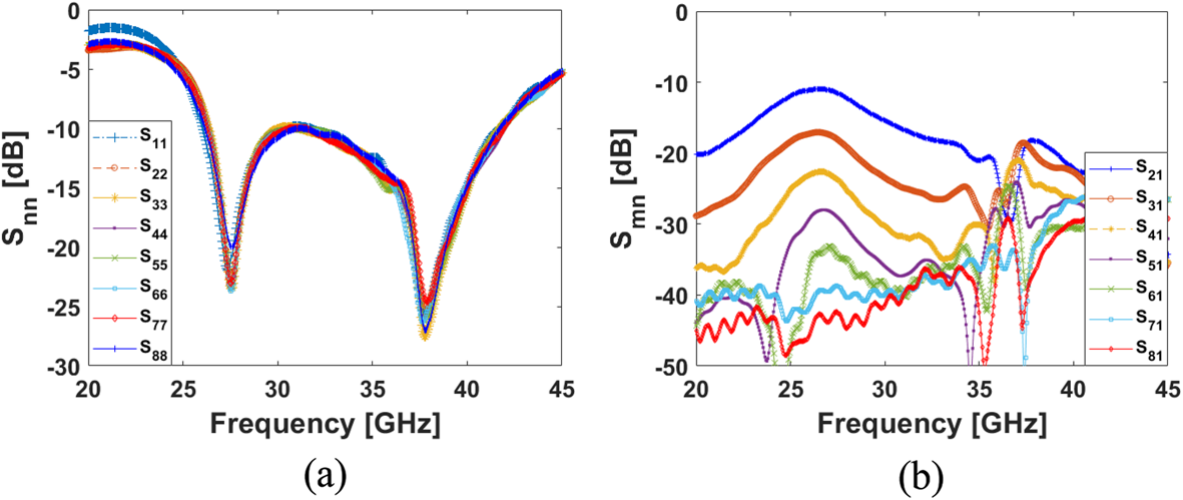
(**a**) S_nn_ (S_11_–S_88_) and (**b**) S_mn_ (S_21_–S_81_) results of the suggested phased array.

In this section, a low-profile MM-Wave phased array with super wideband function is suggested to be incorporated in a shared board of the discussed MIMO smartphone antenna system. It contains eight end-fire loop resonators with a very compact size of W_a_ × L_a_ which can be implemented in the same Rogers substrate material. The configuration of the single-element and its frequency bandwidth are shown in Fig. 24. As shown, the antenna element is highly miniaturized and it is offering a super-wide frequency bandwidth supporting the frequency of 26 to 42 GHz covering several 5G candidate bands such as 26, 28, 32, 36, and 38 GHz. The linear phased array schematic of the introduced design is plotted in Fig. 25. It includes eight loop-dipole elements with low profiles arranged in a linear form. For each antenna element, a discrete feeding port is applied. The phased array S-parameters including S_nn_ (S_11_ to S_88_) and S_mn_ (S_21_ to S_81_) have been provided in Fig. 26a,b. The results indicate that the presented phased array offers ultra- and super-wide bandwidth of 26–42 GHz (16 GHz) with satisfactory couplings (better than − 10 dB). The parameter values (in mm) of the single-element and its suggested linear phased array are as given in Table 3.

**Table 3:** Parameter values of the integrated phased array.

Parameter	L_3_	W_4_	L_5_	W_5_	L_6_
Value (mm)	1.5	3.4	0.2	1.7	0.65
Parameter	W_6_	L_7_	L_a_	W_a_	d_a_
Value (mm)	0.2	0.25	2.25	40	2.8

**Figure 27:**
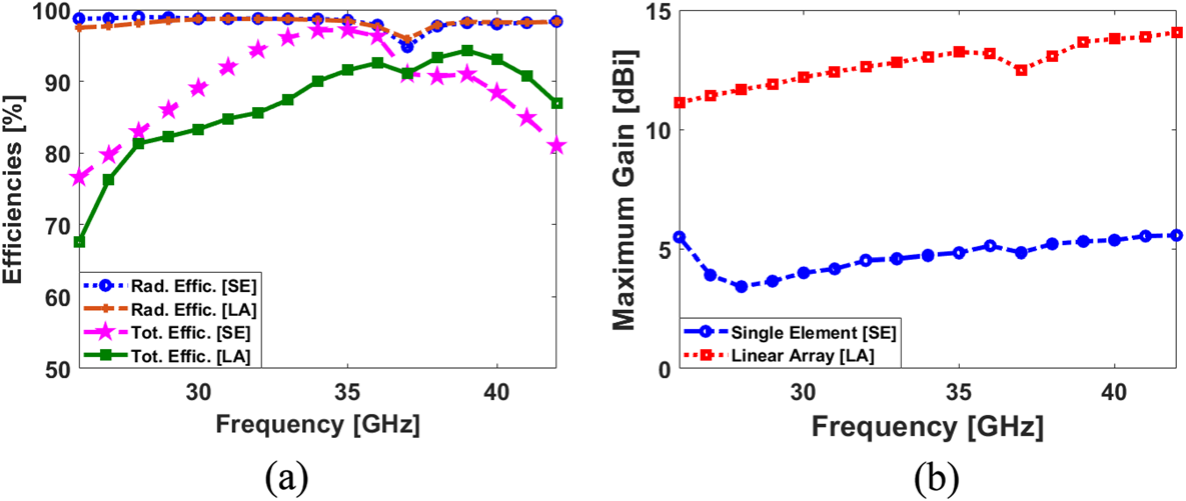
Comparisons of the (**a**) efficiencies and (**b**) maximum gains for the single-element and its linear phased array.

The simulated efficiency results of the single element and the phased array are represented in Fig. 27a. As it is clearly shown, both provide high radiation efficiencies better than 95% over the operation band of 26–42 GHz. In addition, more than 75% and 65% total efficiencies are discovered for the single element and the array, respectively. Moreover, the simulated gain levels of the single and array resonators are compared in Fig. 27b. According to results, the single modified loop offers 3.5–5.2 dBi gain at 26–42 GHz. Alternatively, the linear phased array shows high gains (11–14.5 dBi) over its respective band in which as the frequency increases, the gain improves.

**Figure 28:**
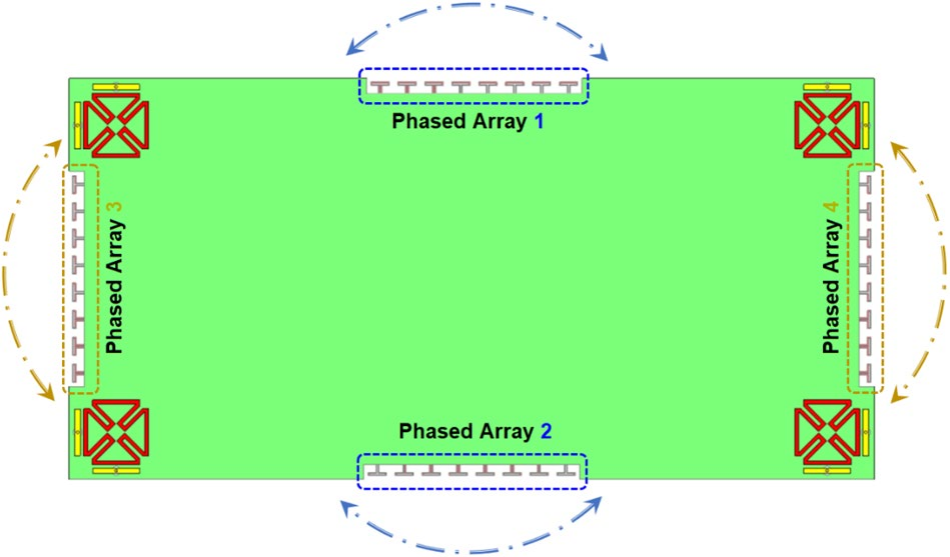
Different placements of the introduced MM-Wave array into the smartphone mainboard.

Figure 28 shows the possible placements of the suggested phased array in the smartphone mainboard. Due to the compact size of the proposed array and also the symmetrical configuration of the smartphone MIMO antenna, the phased array can be placed on different four sides of the mainboard and can provide similar performances. As shown in Fig. 28 the array can easily be integrated onto the PCB in a small area and could provide beam-steerable radiations.

**Figure 29:**
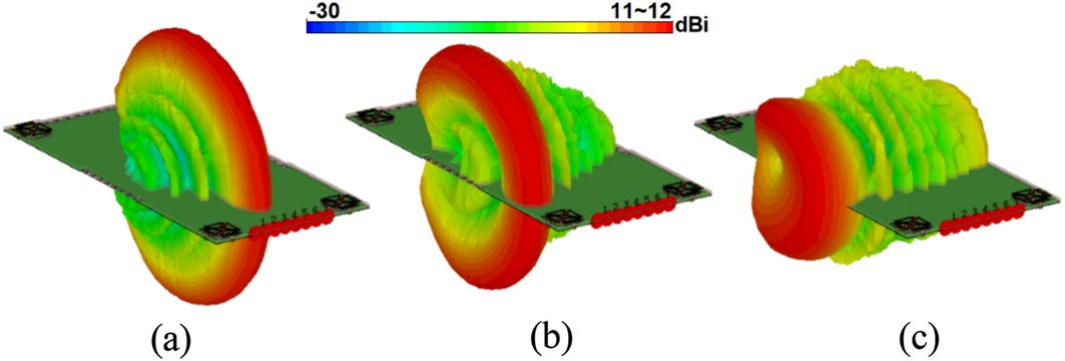
Radiation beam-steering of the phased array (placed at top-edge) at (**a**) 0, (**b**) 30, and (**c**) 60 degrees.

**Figure 30:**
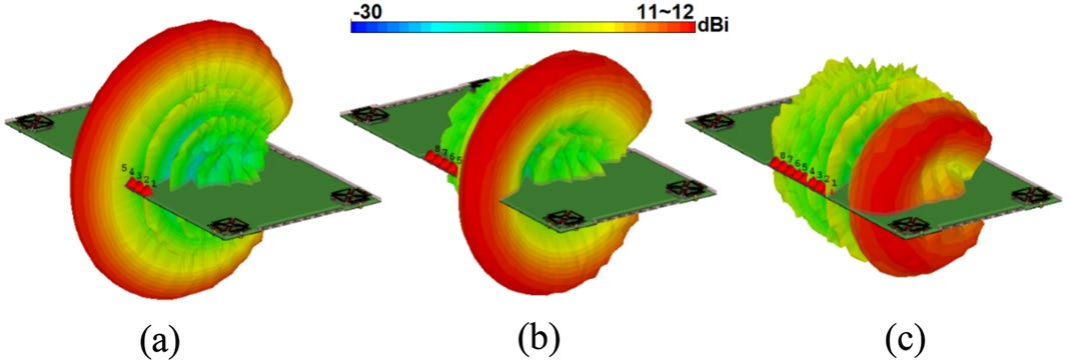
Radiation beam-steering of the phased array (placed at side-edge) at (**a**) 0, (**b**) 30, and (**c**) 60 degrees.

The 3D radiation behaviour of the suggested phased array placed at the top and side edges of the smartphone mainboard with at 30 GHz are represented in Figs. 29 and 30. The plots indicate that the investigated array produces well-defined end-fire radiations with broad scanning. Meanwhile, there have been sufficient gains found from at different steering angles. It is worth mentioning that the array can exhibit similar radiations at minus angles and provide full radiation coverage^43^. Therefore, the suggested phased array can be used in various portable devices because of its numerous promising features, including highly miniaturized profile, super-wide bandwidth, well-defined end-fire radiation, wide beam steering capability, as well as sufficient efficiency/gain levels.

## Conclusion

An eight-resonator MIMO array design formed by employing dual-polarized/self-complementary structures is reported, at the 3.6 GHz 5G band. It is simply constructed on a smartphone board but meanwhile realizes satisfactory properties. Suitable input-impedance characteristics, mutual coupling, and diversity radiations are achieved. As compared with recently reported designs, the proposed smartphone antenna provides improved radiation coverage, less ECC/TARC results, and higher gain/efficiency characteristics. Additionally, it has a planar structure without any ohmic losses, making it an ideal candidate for 5G handheld devices. Meanwhile, the calculated channel capacity and its loss is about 40 and 0.5 bps/Hz for the desired frequency. Simulation and experimental results showed quite good agreement. By appropriately placing the proposed miniaturized resonators, high gain levels and pattern diversity can be achieved. Moreover, a new super-wideband/low-profile MM-wave phased array is suggested to be incorporated in a shared board of the smartphone antenna system. Its critical parameters (frequency response, beam scanning, gain/efficiency) have been examined and sufficient results have been obtained. The suggested MIMO antenna systems can be used in multi-mode/multi-standard 5G cellular communications.

## Data Availability

All Data has been included in study.
