# Publisher Correction: An efficient antenna system with improved radiation for multi-standard/multi-mode 5G cellular communications

**DOI:** 10.1038/s41598-023-32420-y

**Published:** 2023-03-31

**Authors:** Naser Ojaroudi Parchin, Heba G. Mohamed, Karim H. Moussa, Chan Hwang See, Raed A. Abd-Alhameed, Norah Muhammad Alwadai, Ahmed S. I. Amar

**Affiliations:** 1 School of Computing, Engineering and the Built Environment, Edinburgh Napier University, Edinburgh, EH10 5DT UK; 2 Department of Electrical Engineering, College of Engineering, Princess Nourah Bint Abdulrahman University, P.O. Box 84428, Riyadh, 11671 Saudi Arabia; 3 School of Internet of Things, Xi’an Jiaotong-Liverpool University, Suzhou, 215123 Jiangsu Province China; 4 Faculty of Engineering and Informatics, University of Bradford, Bradford, BD7 1DP UK; 5 Department of Physics, College of Engineering, Princess Nourah Bint Abdulrahman University, P.O. Box 84428, Riyadh, 11671 Saudi Arabia; 6 Department of Electronics & Communication, Air Defense College, Alexandria University, Alexandria, Egypt

Correction to: *Scientific Reports* https://doi.org/10.1038/s41598-023-31407-z, published online 13 March 2023

The original version of this Article contained an error in the Funding section.

“The funding was provided by Princess Nourah Bint Abdulrahman University (PNURSP2022TR140).”

now reads:

“The authors extend their appreciation to the Deputyship for Research & Innovation, Ministry of Education in Saudi Arabia for funding this research work through Project Number RI-44-0422.”

The original Article has been corrected.

